# Paget-Schroetter syndrome in the absence of common predisposing factors: a case report

**DOI:** 10.1186/s12959-017-0146-0

**Published:** 2017-08-01

**Authors:** Ramy Ibrahim, Irina Dashkova, Myia Williams, Andrzej Kozikowski, Neeraj Abrol, Anjula Gandhi, Renee Pekmezaris

**Affiliations:** 1 Premier Medical Associates Group Orlando, Florida, USA; 2 Division of Geriatric and Palliative Medicine, Northwell Health, New York, USA; 3 Department of Medicine, Northwell Health, New York, USA; 4 Department of Medicine, Northwell Health, NewYork, USA; 5 Attending Physician, Brookdale Hospital, New York, USA; 6 Internal Medicine Residency Program, Brookdale Hospital, New York, USA; 7 Department of Medicine, Northwell Health, New York, USA

**Keywords:** Deep Vein Thrombosis, Paget-Schrotter Syndrome, Axillary-subclavian venous thrombosis, Pulmonary Embolism, Thrombosis, Upper Extremity Deep Vein Thrombosis

## Abstract

**Background:**

Paget-Schrotter Syndrome (PSS) also known as “effort thrombosis” is a form of primary thrombosis in the subclavian vein at the costoclavicular junction is usually seen in younger patients after repeated strenuous activity of the shoulders and arms. When occurring in younger patients, PSS presents itself with predisposing factors such as unilateral dull, aching pain in the shoulder or axilla and swelling of the arm and hand.

**Case Presentation:**

We report a rare case of unusual left axillo-subclavian vein thrombosis in absence of clear risk factors and a negative hypercoagulable workup in a 36-year-old Hispanic woman who presented with 2 days duration of left upper extremity pain and swelling after a week of heavy exercise in aerobic class. Very few documented cases of primary or spontaneous ASVT in absence of clear factors and in such anatomical location have been previously reported.

The patient was started on strict precautions of left upper extremity immobilization, analgesics in the form of Tylenol 650 mg every 6 h for pain as well as cold compresses. Lovenox 90 mg subcutaneous twice daily (1 mg/kg BID) was started together with warfarin to keep INR 2–3.

**Conclusion:**

In addition to the unusual location in the left upper extremity in our case, the absence of common etiologic factors makes our case of Paget-Schroetter Syndrome a very unique one. Presently, there is a lack of guided management of rare conditions such as our case, or consensus among the sources. Physicians should be aware of this rare disease since untreated conditions may be debilitating for the patient and very costly especially if diagnosed with a delay.

## Background

Paget- Schroetter Syndrome involves axillary-subclavian venous thrombosis (ASVT) associated with strenuous and repeated activity of the upper extremities [1, 2]. PSS is also called “effort” thrombosis denoting that the syndrome often occurs in physically active individuals after unusual strenuous use of the arm and shoulder [3]. In addition, PSS is also referred to as “spontaneous” ASVT, highlighting its often dramatic, unexpected presentation in otherwise healthy young individuals [4, 5]. On average, PSS accounts for at least 10–20% of upper extremity deep thrombosis and at least up to 30–40% of spontaneous axillary-subclavian venous thrombosis [1, 2].

PSS is usually experienced after sporting activities such as swimming, wrestling and gymnastics which often involve vigorous and continued movements of the upper extremity [2]. It is believed that retroversion, hyperabduction and extension of the arm involved in strenuous sporting activities impose undue strain on the subclavian vein; which leads to micro-trauma of the endothelium and activation of the coagulation cascade. Consequently, it is not unusual that PSS usually occurs in the dominant arm of young, healthy and active men [2]. Patients with PSS are customarily symptomatic, with swelling and arm discomfort being the most frequently reported and presented problems, as shown in Table 1 [2, 6–8]. Other symptoms reported include heaviness, redness of arm, cyanosis and dilated, visible veins across the shoulder and upper arm (Urschel’s sign) [2, 6]. Often the symptom onset is either acute or sub-acute, however, occasionally, patients can present with chronic symptoms [2]. In addition, not uncommon symptoms can present as nonspecific and at times mimic those of a muscular strain [2, 9]. Further, a majority of patients have reported a discrete precipitating event of a sports related arm exertion. Similarly, trivial and harmless daily activities can result in PSS [2, 6, 10].

**Table 1:** Manifestations of DVT

Type of Manifestation	
Asymptomatic	
Pain in the arm, neck and shoulder region Diffuse arm swelling	
Discoloration, tenderness and distension on the affected limb	
Visible collaterals on the affected arm Arm discoloration and palpable vessels	

Complications also seen in patients with PSS include pulmonary embolism (PE), post thrombotic syndrome and recurrent thrombosis [2, 6, 7]. It is important to note there are mixed reports of lower incidence of PE in upper extremity DVT when compared with lower extremity DVT and catheter related UEDVT [2, 8, 11, 12]. Regardless, health practitioners should bear in mind that the risk of PE in patients with PSS is real and significant [2, 6, 7, 13, 14].

We report a rare case of unusual left axillosubclavian vein thrombosis in absence of clear risk factors and a negative hypercoagulable workup in a 36-year-old Hispanic woman who presented with 2 days duration of left upper extremity pain and swelling after a week of heavy exercise in aerobic class. Very few documented cases of primary or spontaneous ASVT in absence of clear factors and in such anatomical location have been previously reported.

## Case presentation

A 36 year old apparently healthy Hispanic female presented to the emergency department (ED) with a 4 days history of left upper extremity pain dull aching in nature and tenderness to palpation after a week of strenuous activity. Her background history was without any significant family history or risk factors. Physical examination revealed a moderately nourished, well-built female, not in acute distress except for marked pain in left extremity. No other abnormality was detected on physical examination.

A complete blood count was done as part of a routine examination. Doppler studies of the four extremities was done in ED which showed left axillosubclavian acute DVT. Laboratory results are presented in Table 2 and Table 3 below. Secondary to the elevated D-Dimers patient underwent CT chest and pulmonary angiography to rule out pulmonary extension or pulmonary embolism (PE). The CT results confirmed the presence of left axillo-subclavian venous thrombosis; however, there was no evidence of PE.

**Table 2:** Initial Admission Laboratory Results

Parameter	Values	Range
WBC	6.2	4–11
RBC	3.63	3.8–5.3
HCT	32	37–51
MCV	88.1	80–101
MCHC	30.8	27–32
RDW	12.6	11.5–14.5
Platelet	244	130–450
D-dimer	2203	0–278
BUN	15	8–25
Creatinine	0.7	0.5–1.04
Sodiun	138	133–145
Potassium	4.4	3.5–5.5
Chloride	101	96–101
Carbon dioxide	27	22–28
Calcium	9.4	8.5–10.5
Antithrombin III	113	85–120
Protein C	110	70–180
Lupus anticoagulant	45	<40
Proteins	94	60–140

**Table 3:** Risk factors for Paget-Schroetter Syndrome

Risk Factors	
Physical activity involving hyperabduction of the shoulder, as seen in weight lifters	
Motions often associated with tennis players and baseball pitchers	
Vigorous exercise of the neck and upper extremity muscles	
Overdeveloped anterior scalene muscle Rudimentary first rib	
Presence of cervical rib	
Congenital band between first and second ribs Fracture of the clavicle with callus formation	
Apical tumors of the superior sulcus of the lung (Pancoast tumor)	
Thoracic outlet syndrome	

Futhermore, a CT chest was done and results showed there were no anatomical abnormalities obstructing thoracic outlet. It is possible that strenuous physical activity with temporary obstruction of the thoracic outlet while patient was training her upper body has triggered and likely temporary dehydration caused by extensive sweating during physical training further contributed to the thrombotic event.

The patient was started on strict precautions of left upper extremity immobilization, analgesics in the form of Tylenol 650 mg every 6 h for pain as well as cold compresses. Lovenox 90 mg subcutaneous twice daily (1 mg/kg BID) was started together with warfarin to keep INR 2–3. On the third day of hospitalization the therapeutic INR was reached and patient was discharged.

Additional workup to exclude hypercoagulable state in the form of antiphospholilpid antibody, factor V, Leyden, protein S and C and antithrombin III were within normal levels with no gross abnormality suggestive of thrombophilic state. Catheter-guided thrombolysis was considered with option to transfer patient to specialized center since this type of treatment was not available at the described facility. However patient was not willing to relocate and preferred to be treated at the same facility she was admitted to originally knowing that other type of treatment is available at the other center.

Two months after discharge, patient came for follow up. Doppler study showed that there were no blood clots in axillosubclavian vessels and all blood work was within normal limits including D-Dimers of 177 and the patient clinically asymptomatic.

## Discussion and conclusions

In this report, we have noticed a relatively uncommon presentation of DVT in the upper extremity in absence of any common risk factor.

Stress thrombosis or primary ASVT syndrome can occur in apparently healthy individuals without Virchow’s triad or other thrombosis enhancing risk factors. How can thrombosis occur in patients without any apparent predisposition? The exact mechanism of this is not well understood, however, it may be related to minor thoracic inlet abnormalities together with strenuous physical activity which defies the state of stasis in Virchow’s triad. The abnormalities of the thoracic outlet are often bilateral and predispose to eventual thrombosis of both venous systems [15]. In addition, chronic compression of the vein can cause perivenous fibrosis, which may result in partial venous obstruction despite surgical correction of the compressing lesion [15, 16]. In our case 2 months after hospitalization patient treated with anticoagulation with Warfarin came for a follow up. Her symptoms were resolved so to were blood clot in axillosubclavian veins.

Condition management should include differential diagnosis such as cellulitis, lymphedema, neoplastic compression of veins, traumatic muscle injury, and thrombosis of superficial veins. A detailed imaging panel including dopplers, CT angiography and even MRI/MRA must be considered. Laboratory testing should include CBC with special focus on platelet count to exclude other etiologies, as well as a complete panel of hypercoagulable work up to exclude secondary causes [17]. Should clinical picture suggest ASVT, the best test to perform first is the duplex sonogram. It is inexpensive, highly sensitive, specific and non-invasive way to diagnose condition without delay of treatment.

Patients may have good prognosis and better outcome of thrombolytic therapy, if ASVT was diagnosed early, and extent of damages is limited. In case of skeletal abnormality, compressing venous structures as a cause of ASVT, surgical intervention such as rib or clavicle resection may be needed. Rehabilitation and physical therapy are an important part of patient’s management, even more so in case of purely muscular causes of venous blood flow obstruction.

Acute deep venous thrombosis is a very common problem affecting up to one in every thousand Americans; however, upper extremity presentation is much less common. Excluding surgical causes, catheter-induced upper extremity venous thrombosis becomes a rare presentation. However by further excluding secondary causes only very few cases of primary upper extremity DVT or PSS have been reported. As a result, more research is needed in ASVT. Presently, there is a lack of guided management of rare conditions such as our case, or consensus among thesources. Physicians should be aware of this rare disease since untreated conditions may be debilitating for the patient and very costly especially if diagnosed with a delay. Absence of anatomical obstruction and favorable outcome after conventional treatment makes this case unique.

## Abbreviations

ASVT: Axillary-subclavian venous thrombosis
CBC: Complete Blood Clot
DVT: Deep Vein Thrombosis
ED: Emergency department
INR: International Normalised Ratio
MRA: Magnetic Resonance Angiogram
MRI: Magnetic Resonance Imaging
PE: Pulmonary embolism
PSS: Paget-Schrotter Syndrome
UEDVT: Upper Extremity Deep Vein Thrombosis
